# Feasibility of cell-based therapy combined with descemetorhexis for treating Fuchs endothelial corneal dystrophy in rabbit model

**DOI:** 10.1371/journal.pone.0191306

**Published:** 2018-01-16

**Authors:** Naoki Okumura, Daiki Matsumoto, Yuya Fukui, Masataka Teramoto, Hirofumi Imai, Tetta Kurosawa, Tomoki Shimada, Friedrich Kruse, Ursula Schlötzer-Schrehardt, Shigeru Kinoshita, Noriko Koizumi

**Affiliations:** 1 Department of Biomedical Engineering, Faculty of Life and Medical Sciences, Doshisha University, Kyotanabe, Japan; 2 Department of Ophthalmology, University of Erlangen-Nürnberg, Erlangen, Germany; 3 Department of Frontier Medical Science and Technology for Ophthalmology, Kyoto Prefectural University of Medicine, Kyoto, Japan; Cedars-Sinai Medical Center, UNITED STATES

## Abstract

Corneal transparency is maintained by the corneal endothelium through its pump and barrier function. Severe corneal endothelial damage results in dysregulation of water flow and eventually causes corneal haziness and deterioration of visual function. In 2013, we initiated clinical research of cell-based therapy for treating corneal decompensation. In that study, we removed an 8-mm diameter section of damaged corneal endothelium without removing Descemet’s membrane (the basement membrane of the corneal endothelium) and then injected cultured human corneal endothelial cells (CECs) into the anterior chamber. However, Descemet’s membrane exhibits clinically abnormal structural features [i.e., multiple collagenous excrescences (guttae) and thickening] in patients with Fuchs endothelial corneal dystrophy (FECD) and the advanced cornea guttae adversely affects the quality of vision, even in patients without corneal edema. The turnover time of cornea guttae is also not certain. Therefore, we used a rabbit model to evaluate the feasibility of Descemet’s membrane removal in the optical zone only, by performing a small 4-mm diameter descemetorhexis prior to CEC injection. We showed that the corneal endothelium is regenerated both on the corneal stroma (the area of Descemet’s membrane removal) and on the intact peripheral Descemet’s membrane, based on the expression of function-related markers and the restoration of corneal transparency. Recovery of the corneal transparency and central corneal thickness was delayed in areas of Descemet’s membrane removal, but the cell density of the regenerated corneal endothelium and the thickness of the central corneal did not differ between the areas with and without residual Descemet’s membrane at 14 days after CEC injection. Here, we demonstrate that removal of a pathological Descemet’s membrane by a small descemetorhexis is a feasible procedure for use in combination with cell-based therapy. The current strategy might be beneficial for improving visual quality after CEC injection as a treatment for FECD.

## Introduction

The cornea, a transparent tissue located at the front of the eye, allows light to enter the eye. Corneal transparency is maintained by the corneal endothelium through a pump and barrier function. The corneal endothelium, which is a monolayer cell sheet located at the anterior chamber aspect of the cornea, regulates the amount of water in the corneal stroma [1]. Therefore, severe corneal endothelial damage results in dysregulation of water flow and eventually leads to corneal haziness.

The only therapeutic option for treating this corneal endothelial decompensation is corneal transplantation using donor corneas [2]. The use of penetrating keratoplasty, in which a full thickness cornea is replaced by a donor cornea, was first documented in 1906, and this procedure has been widely performed [3]. Strategies for the replacement of damaged corneal endothelium that do not involve a full-thickness corneal transplantation, such as Descemet’s stripping endothelial keratoplasty (DSEK) and Descemet’s membrane endothelial keratoplasty (DMEK), were introduced in 2000s, and these two have gained worldwide prevalence [4–8]. However, surgical challenges, long-term cell loss after transplantation, and a shortage of donor cornea tissue still represent major problems associated with corneal transplantation [2, 9].

Accordingly, many researchers have devoted their efforts to the development of alternative techniques of regenerative medicine to overcome those problems [10–14]. Our group has found that inhibition of Rho kinase (ROCK) signaling enhances corneal endothelial cell (CEC) adhesion to a substrate [15], and we reported the usefulness of ROCK inhibitor in cell-based therapy with CECs [16, 17]. In rabbit and monkey corneal endothelial dysfunction models, injection of cultured CECs in combination with a ROCK inhibitor resulted in corneal endothelium regeneration [16, 17]. In 2013, we initiated clinical research into cell-based therapy at the Kyoto Prefectural University of Medicine (Clinical trial registration: UMIN000012534) [18]. In that clinical study, we removed an 8-mm diameter portion of the damaged corneal endothelium without removing Descemet’s membrane (the basement membrane of the corneal endothelium), and then injected cultured human CECs, in combination with a ROCK inhibitor, into the anterior chamber. Our assumption was that the remaining basement membrane would provide ideal conditions for CEC adhesion and survival.

Corneal endothelial cell ablation without removal of Descemet’s membrane has, however, both advantages and disadvantages in the clinical setting. Although the presence of a basement membrane may support efficient adhesion of injected cultured CECs and may provide ideal conditions for cell fate and viability, Descemet’s membrane of patients with Fuchs endothelial corneal dystrophy (FECD) exhibits a clinically abnormal structure, including multiple collagenous excrescences (guttae) and thickening [19, 20]. Recent clinical studies have showed that guttae affect the quality of vision, even in patients without corneal edema, although guttae have been previously assumed not to affect visual quality [21]. Wacker and colleagues reported that anterior and posterior corneal high-order aberrations (HOAs) and backscatter are higher even in early stages of FECD than in non-FECD subjects [22]. They suggested that increased posterior HOAs due to guttae are responsible for decreased vision, even in the absence of clinically detectable corneal edema [22]. Watanabe and colleagues also showed that the presence of guttae negatively correlates with clinical parameters for quality of vision, such as corrected distance visual acuity, letter contrast sensitivity, and stray light [23]. They assumed that guttae induce irregularity and posterior corneal opacity, resulting in an increase in forward light scatter [23].

Thus, we were motivated to modify our surgical protocol for cultured CEC injection by removing the pathological Descemet’s membrane in patients with FECD during surgery. In this study, we used a rabbit model to evaluate the feasibility of Descemet’s membrane removal in the optical zone prior to CEC injection.

## Materials and methods

### Ethics statement

Human corneas were handled in accordance with the tenets set forth in the Declaration of Helsinki. Experiments using human tissue samples were approved by the Institutional Review Board of the Medical Faculty of the University of Erlangen-Nürnberg (No. 4218-CH). Informed verbal consent was acquired from the donors or their relatives. The rabbit experiments were performed at Doshisha University (Kyoto, Japan), according to the protocol approved by Doshisha University Animal Experiment Committee (Approval No. A17066).

### Human corneal specimens

Corneal specimens for histopathologic examination were obtained during penetrating keratoplasty, performed in 1995, from patients with FECD with aquiring verval informed consent (n = 5, mean age 73.3 ± 7.9 years). Corneal specimens from normal donor eyes (n = 5, mean age 74.5 ± 5.7 years) with no history of ocular disease but deemed unsuitable for transplantation, were procured from the Erlangen Cornea Bank. Donor corneas were fixed within 15 hours after death in 4% paraformaldehyde/1% glutaraldehyde in 0.1M phosphate buffer (pH 7.4) and processed for light and transmission electron microscopy according to standard protocols.

### Rabbit Corneal Endothelial Cell (RCEC) culture

Ten rabbit eyes were purchased from the Funakoshi Co., Ltd. (Tokyo, Japan). The rabbit corneal endothelial cells (RCECs) were cultivated as described previously [16, 24]. Briefly, Descemet’s membrane with RCECs was stripped and incubated in 0.6 U/mL of Dispase II (Roche Applied Science, Penzberg, Germany). The RCECs isolated from the Descemet’s membrane were seeded in one well of a 6-well plate coated with FNC Coating Mix^®^ (Athena Environmental Sciences, Inc., Baltimore, MD). The RCECs were cultured in a growth medium composed of Dulbecco’s modified Eagle’s medium (DMEM, Life Technologies Corp., Carlsbad, CA) supplemented with 10% fetal bovine serum (FBS), 50 U/mL penicillin, 50 μg/mL streptomycin, and 2 ng/mL fibroblast growth factor 2 (Life Technologies Corp.). Cultivated RCECs were used at passages 2 through 3 for all experiments.

### Injection of RCECs into a corneal endothelial dysfunction model with or without descemetorhexis

Eighteen rabbits were used in this experiment. One eye of each rabbit was used for the experiments to avoid blindness in both eyes. The rabbit corneal endothelial dysfunction model was created as described previously [16, 24]. Briefly, the lens was removed to deepen the anterior chamber. One week after this lens removal, the corneal endothelium was mechanically scraped from Descemet’s membrane with a 20-gauge silicone needle (Soft Tapered Needle; Inami & Co., Ltd., Tokyo, Japan). Total removal of corneal endothelium was confirmed by 0.2% trypan blue (Sigma-Aldrich, St. Louis, MO, USA) staining. A 4 mm diameter continuous circular descemetorhexis (CCD) was performed in 6 eyes (CCD (+) group). A total of 5.0×10^5^ RCECs, suspended in 200 μl DMEM supplemented with 100 μM Y-27632 ROCK inhibitor (Wako Pure Chemical Industries, Ltd.), was injected into the anterior chamber of the corneal endothelial dysfunction model and the animals were kept in the face-down position for 3 hours under general anesthesia. RCECs were injected into 6 eyes of the CCD (+) group (the corneal endothelium was totally removed and a 4 mm CCD was performed) and 6 eyes of the CCD (-) group (corneal endothelium was totally removed and CCD was not performed). As a control for the corneal endothelial dysfunction model, 6 eyes had the corneal endothelium was totally removed, but CCD was not performed and no RCECs were injected.

The anterior segments of the eyes were evaluated by slit-lamp microscopy and with a Pentacam^®^ (OCULUS Optikgeräte GmbH, Wetzlar, Germany) instrument for 2 weeks. Central corneal thickness was determined with an ultrasound pachymeter (SP-2000; Tomey, Nagoya, Japan), and the mean of 10 measured values was calculated (up to a maximum thickness of 1200 μm, the instrument’s maximum reading). The corneal endothelium was evaluated by contact specular microscopy (Konan scanning slit specular microscope, Konan Medical, Nishinomiya, Japan). Intraocular pressure was determined with a Tonovet^®^ (icare Finland, Vantaa, Finland) instrument.

### Periodic acid-Schiff (PAS) staining and immunocytochemistry

Corneas were fixed in 4% buffered paraformaldehyde and processed for paraffin embedding. Sections cut 5 μm thick were stained with PAS according to standard protocols. Rabbit corneal specimens were fixed in 4% formaldehyde and incubated for 30 minutes in 1% bovine serum albumin (BSA) to block nonspecific binding. Corneas were examined by actin staining performed with a 1:400 dilution of Alexa Fluor^®^ 546-conjugated phalloidin (Life Technologies Corp.). For immunohistochemical analyses, specimens were incubated overnight at 4°C with primary antibodies against Na^+^/K^+^-ATPase (1:300, Upstate Biotechnology, Lake Placid, NY), ZO-1 (1:300, Life Technologies Corp.), and N-cadherin (1:300, BD Biosciences, San Jose, CA). The specimens were then incubated for 2 hours at 4°C with secondary antibody, Alexa Fluor^®^ 488-conjugated goat anti-mouse (1:1000, Life Technologies Corp.). Nuclei were stained with DAPI (Vector Laboratories, Burlingame, CA). The slides were examined with a fluorescence microscope (TCS SP2 AOBS; Leica Microsystems, Wetzlar, Germany).

### Transmission electron microscopy

For transmission electron microscopy, cornea specimens were fixed in 4% paraformaldehyde/1% glutaraldehyde in 0.1M phosphate buffer, postfixed in 2% buffered osmium tetroxide, dehydrated in graded alcohol concentrations, and embedded in epoxy resin according to standard protocols. Ultrathin sections were stained with uranyl acetate and lead citrate and examined with a transmission electron microscope (EM 906E; Carl Zeiss NTS GmbH, Oberkochen, Germany).

Alternatively, corneal buttons were washed in 0.1M Sorensen phosphate buffer, processed through 0.5% uranyl acetate en bloc staining, and then dehydrated using an ascending ethanol series. Samples were transferred to propylene oxide and were embedded in Araldite CY212 resin. Ultrathin sections were collected on uncoated G300 copper grids and stained with 1% aqueous phosphotungstic acid and uranyl acetate. Sections were examined with a transmission electron microscope (JEOL 1010; JEOL, Tokyo, Japan) equipped with a charge-coupled device camera (Orius SC1000; Gatan, Pleasanton, CA).

### Statistical analysis

The statistical significance (*P*-value) for mean values in two-sample comparisons was determined with the Student’s t-test. The statistical significance of comparisons of multiple sample sets was analyzed with the Dunnett’s multiple-comparisons test. Results were expressed as mean ± SEM.

## Results

### Histology of Descemet’s membrane of FECD patients

**Fig 1:**
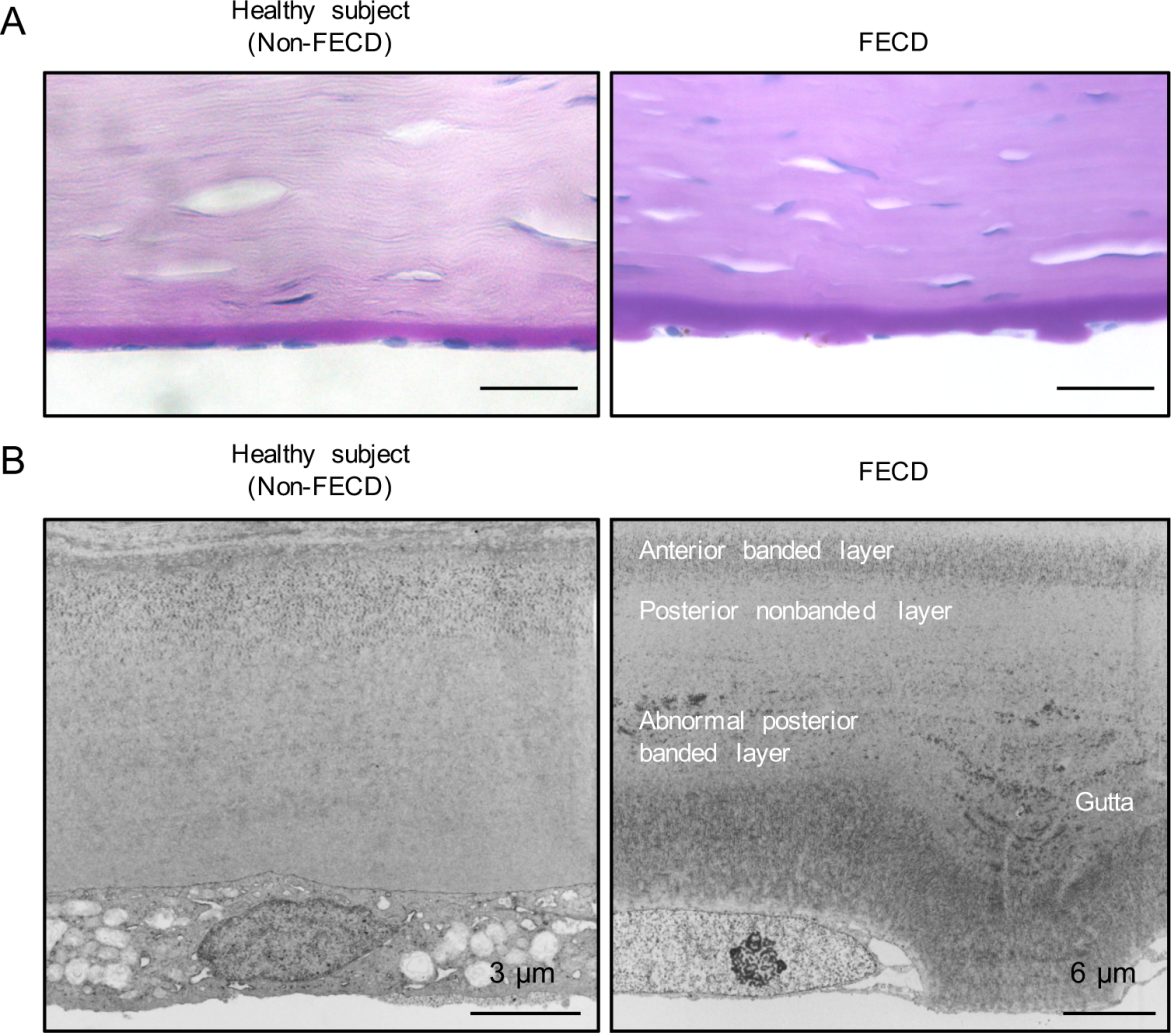
Histology of excrescences of Descemet’s membrane in patients with FECD. (A) Representative PAS staining images of a healthy donor cornea (left).Representative PAS staining images of a cornea obtained from a patient with FECD. Excrescences, which are clinically called guttae”, were observed on Descemet’s membrane of the patient with FECD. Scale bar: 50 μm (right). (B) Ultra structural analysis of Descemet’s membrane of non-FECD donor cornea was observed using TEM. Scale bar: 3 μm (left). Ultrastructural TEM analysis of Descemet’s membrane of a cornea obtained from a patient with FECD. Flattened CECs adhered to the excrescences on Descemet’s membrane. Scale bar: 6 μm (right).

PAS staining showed the presence of abnormal excrescences, which are clinically called guttae, on the posterior aspect of Descemet’s membrane in patients with FECD (Fig 1A, right). No similar excrescences were observed in non-FECD donor corneas (Fig 1A, left). Descemet’s membrane was also thicker in corneas from patients with FECD in non-FECD donor corneas, which is consistent with previous reports on abnormal extracellular matrix accumulation by pathological CECs [25, 26]. TEM further showed that attenuated CECs adhered to the outer aspect of the excrescences on Descemet’s membrane in FECD eyes (Fig 1B, right), while CECs formed a sheet-like monolayer on a smooth surface of Descemet’s membrane in non-FECD eyes (Fig 1B, left).

### RCEC injection in the rabbit corneal endothelial dysfunction model with removal of Descemet’s membrane

**Fig 2:**
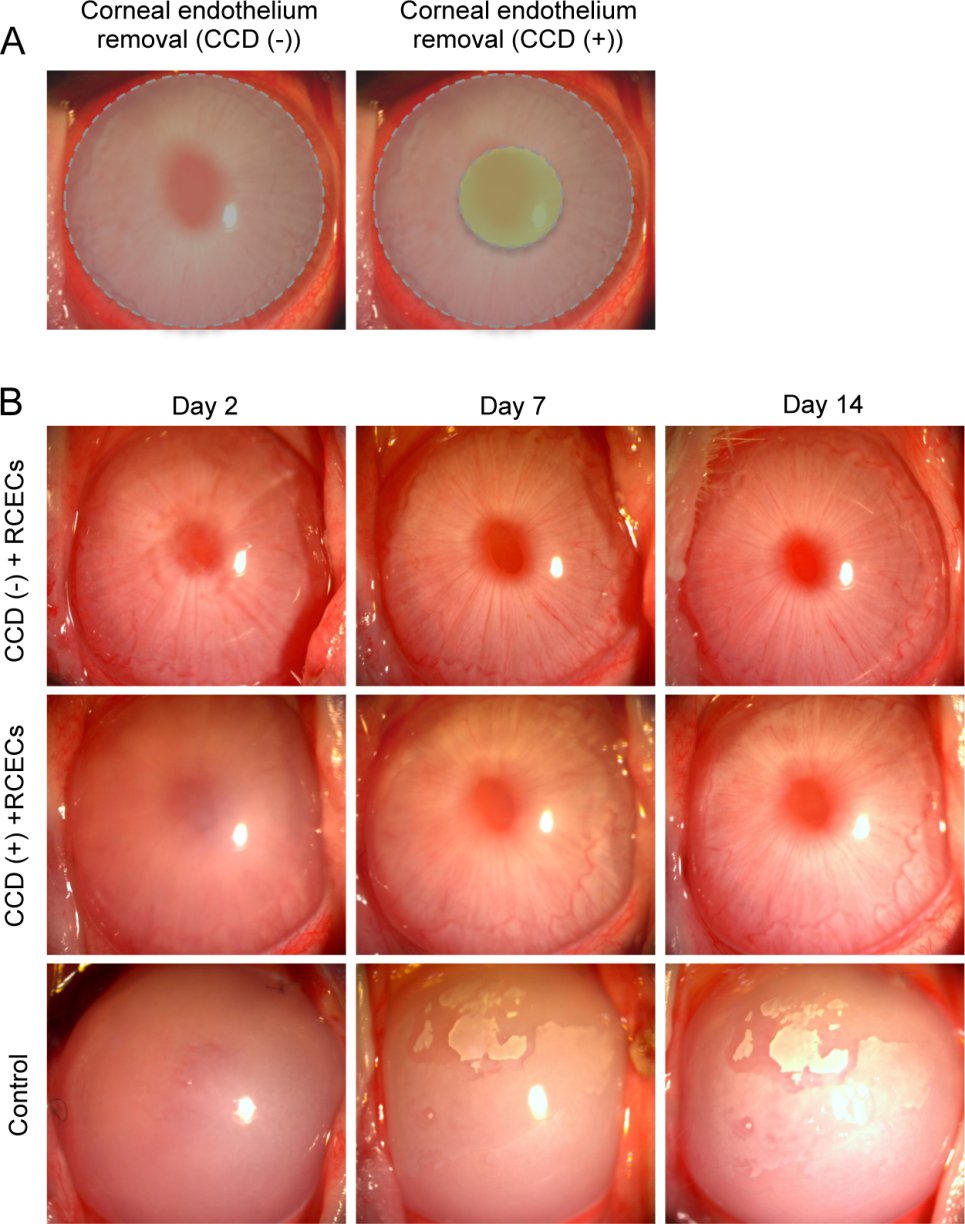
Rabbit corneal endothelium cell (RCEC) injection in the rabbit corneal endothelial dysfunction model with removal of a small central part of Descemet’s membrane. (A) The corneal endothelium was totally scraped from the Descemet’s membrane with a 20-gauge silicone needle, leaving the remaining Descemet’s membrane intact. These eyes were used as the circular descemetorhexis (CCD) (-) group (left). As the CCD (+) group, a 4-mm diameter of Descemet’s membrane was removed by CCD following total corneal endothelium removal. The gray area indicates the area where the corneal endothelium was removed. The green area indicates the area where Descemet’s membrane was removed. (B) A total of 5.0×10^5^ RCECs, suspended in 200 μl of medium supplemented with 100 μM Y-27632 ROCK inhibitor, was injected into the anterior chamber of the (CCD (-) and CCD (+) groups) (n = 6). Six eyes from which the corneal endothelium was totally removed and Descemet’s membrane remained intact were used as controls. Corneal transparency was restored by intracameral injection of RCECs both in CCD (-) and CCD (+) groups, while control eyes exhibited hazy corneas due to corneal endothelial dysfunction.

In our previous rabbit corneal endothelial dysfunction model, we removed the entire corneal endothelium by mechanical scraping, leaving Descemet’s membrane intact (the CCD- model in the present study) (Fig 2A, left). In the current study, our intention was to use a rabbit model to evaluate the feasibility of removing Descemet’s membrane in the central cornea only and to couple this with cell-based therapy as a treatment for FECD. For this purpose, we removed a 4-mm diameter of Descemet’s membrane in the optical zone by CCD following total corneal endothelium ablation (the CCD+ model) (Fig 2A, right). Slitlamp microscopy showed that corneal transparency was restored two days after injection of cultured RCECs in the CCD (-) model and the cornea was completely transparent after 7 days (Fig 2B, upper panel). In the CCD (+) model, corneal transparency recovered equally well as in the CCD (-) model 14 days after cultured RCEC injection. However, the time to restore corneal clarity was longer in the CCD (+) model than in the CCD (-) model (Fig 2B, middle panel). By contrast, control eyes without injection of cultured RCECs showed corneal haze due to corneal endothelial dysfunction throughout the entire 14-day experimental period (Fig 2B, lower panel).

**Fig 3:**
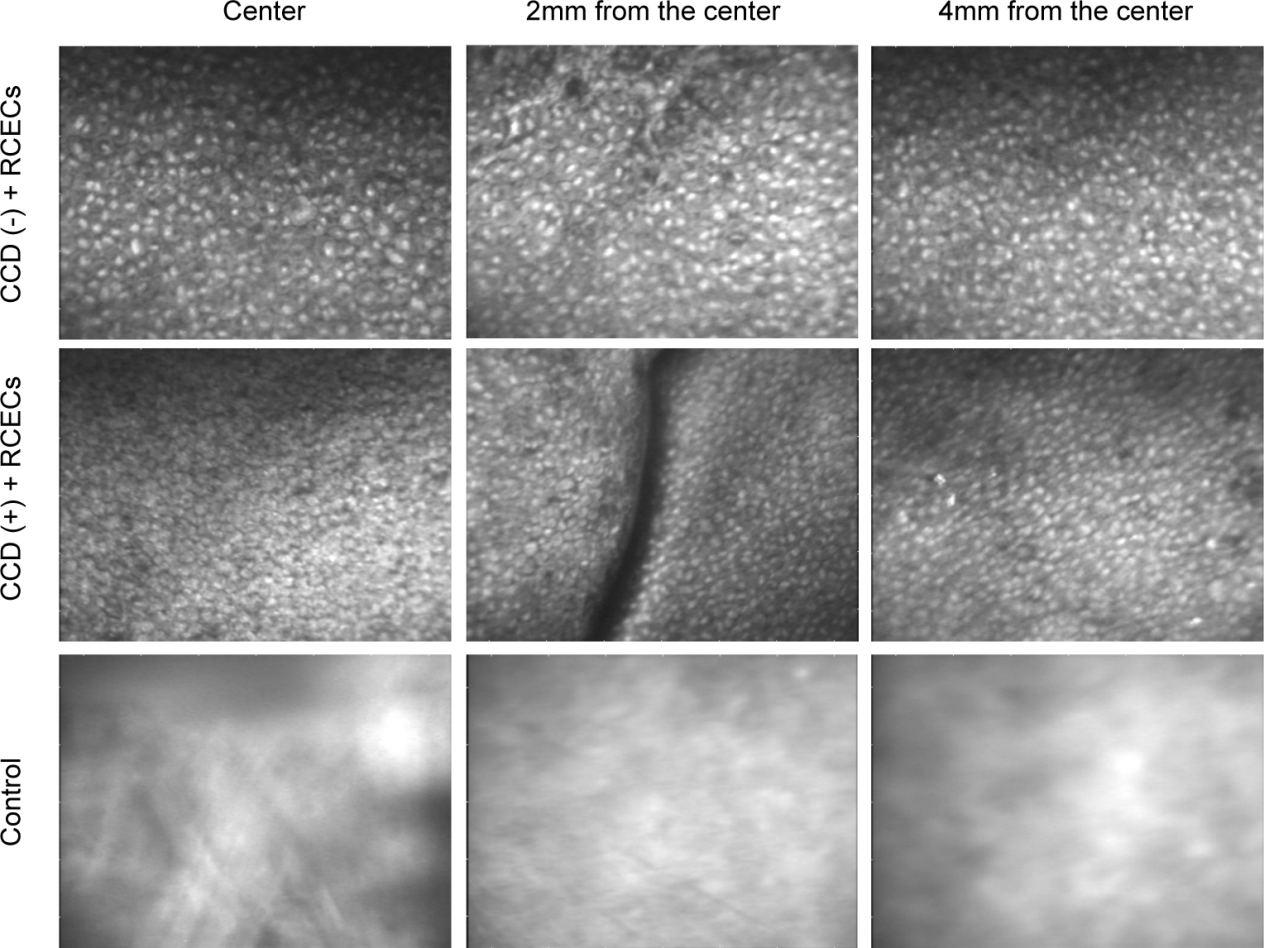
Evaluation of restored corneal endothelium by contact specular microscopy. Restored corneal endothelium was evaluated using contact specular microscopy 14 days after rabbit corneal endothelium cells (RCECs) injection. A representative image showed that monolayer corneal endothelium was restored in the eyes without circular descemetorhexis (CCD) (-) model) (upper panel). In the eyes with circular descemetorhexis (CCD (+) model), hexagonal monolayer corneal endothelium was observed throughout the center to the periphery. The edge of the CCD was observed, and cell density and morphology were similar for the regenerated RCECs directly on the corneal stroma and on Descemet’s membrane. Cell density is likely to be similar regardless the area (middle panel). No corneal endothelial image was observed in the control eyes, which were not injected RCECs (Fig 3, lower panel).

We next examined the restored corneal endothelium using contact specular microscopy 14 days after RCEC injection. Representative specular microscopic images of the CCD (-) model showed a regular monolayer of corneal endothelium with a homogenous cell density (Fig 3, upper panel). The CCD (+) model showed a monolayer of hexagonal corneal endothelial cells extending from the center to periphery, across the edge of the CCD. The cell density and morphology of the regenerated RCECs were similar in the central corneal stroma and on the peripheral Descemet’s membrane (Fig 3, middle panel). No corneal endothelial image could be obtained in control eyes that had not undergone RCEC injection (Fig 3, lower panel).

### Histological analysis of restored corneal endothelium

**Fig 4:**
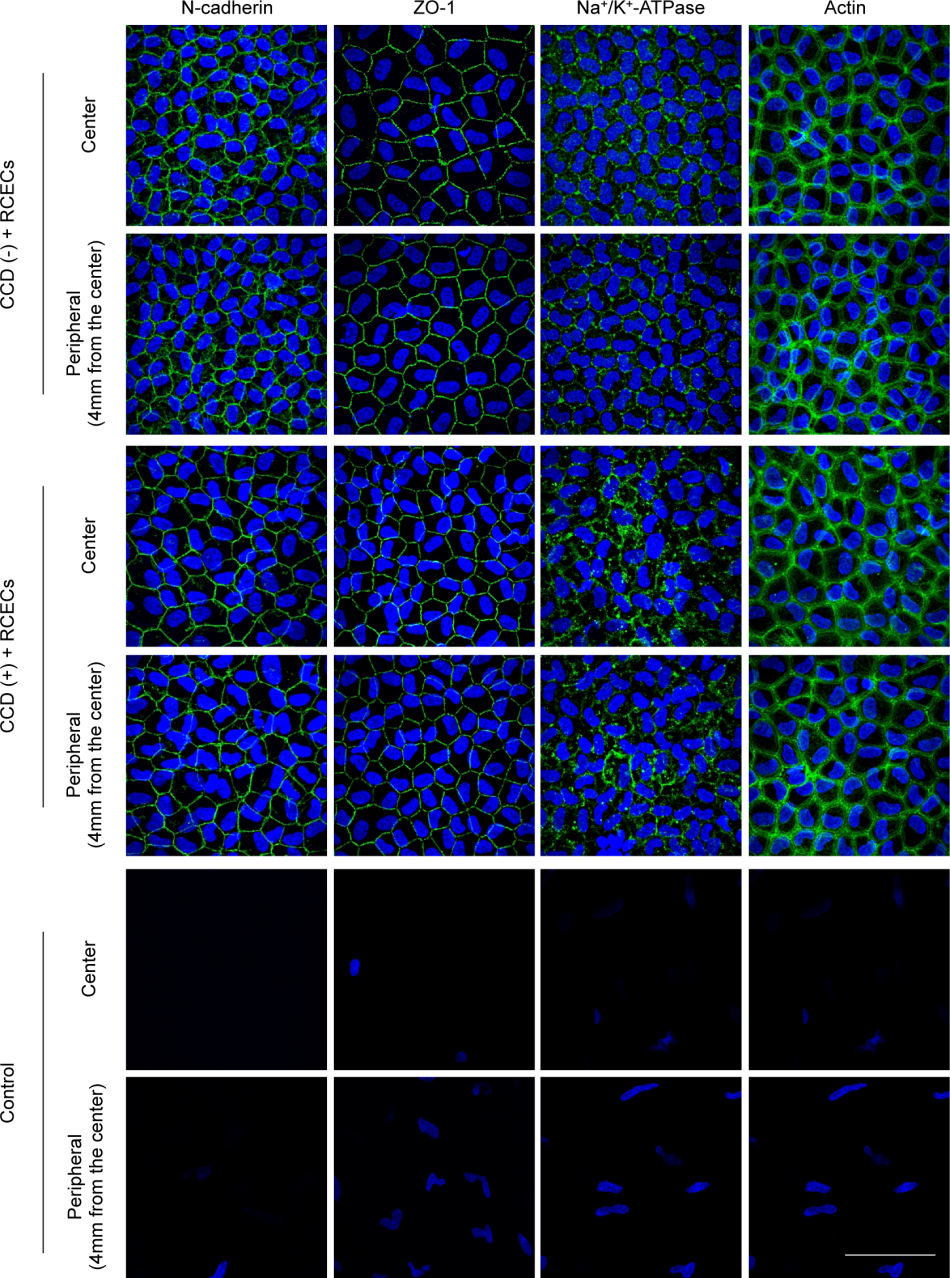
Histological assessment of corneal endothelium regenerated by injection of rabbit corneal endothelium cells (RCECs). Regenerated corneal endothelium was evaluated by immunofluorescent staining 2 weeks after RCECs injection. The eyes without circular descemetorhexis ((CCD) (-) model) showed barrier function-related markers (N-cadherin and ZO-1) and the pump function-related marker Na^+^/K^+^-ATPase at the cell-cell border. Expression patterns of those markers were similar in both the central and the peripheral areas. Actin staining showed that restored corneal endothelium had a polygonal contact-inhibited phenotypic pattern in both the central and peripheral areas (1st to 2nd lines). In the eyes with circular descemetorhexis (CCD (+) group), N-cadherin, ZO-1, and Na^+^/K^+^-ATPase were expressed at the cell-cell border. The expression patterns of these markers in the center, where Descemet’s membrane was removed, was almost the same as in the peripheral area where Descemet’s membrane was not removed. Actin staining showed that the restored corneal endothelium was morphologically similar in both the central and peripheral areas (3rd to 4th lines). In the control eyes, which were not injected with RCECs, almost no cells were observed on Descemet’s membrane (5th to 6th lines). Scale bar: 50 μm.

Immunofluorescence staining demonstrated the presence of the barrier function-related markers (N-cadherin and ZO-1) and pump function-related marker Na^+^/K^+^-ATPase along the cell-cell borders in the CCD (-) model. The expression patterns of these markers were similar in both the central and peripheral areas. Actin staining showed that the restored corneal endothelium exhibited a polygonal, contact-inhibited phenotype in both the central and peripheral areas (Fig 4, 1st to 2nd lines). In the CCD (+) model, N-cadherin, ZO-1, and Na^+^/K^+^-ATPase were also expressed along the cell-cell borders. Notably, the expression patterns of these markers were comparable in the central regions lacking Descemet’s membrane and in the peripheral regions that retained intact Descemet’s membrane. Actin staining demonstrated that the restored corneal endothelium was morphologically similar in both the center and the periphery (Fig 4, 3rd to 4th lines). In the control eyes without RCEC injection, almost no cells were observed on the Descemet’s membrane without inflammatory cells (Fig 4, 5th to 6th lines).

### Evaluation of the effect of CCD on clinical parameters

**Fig 5:**
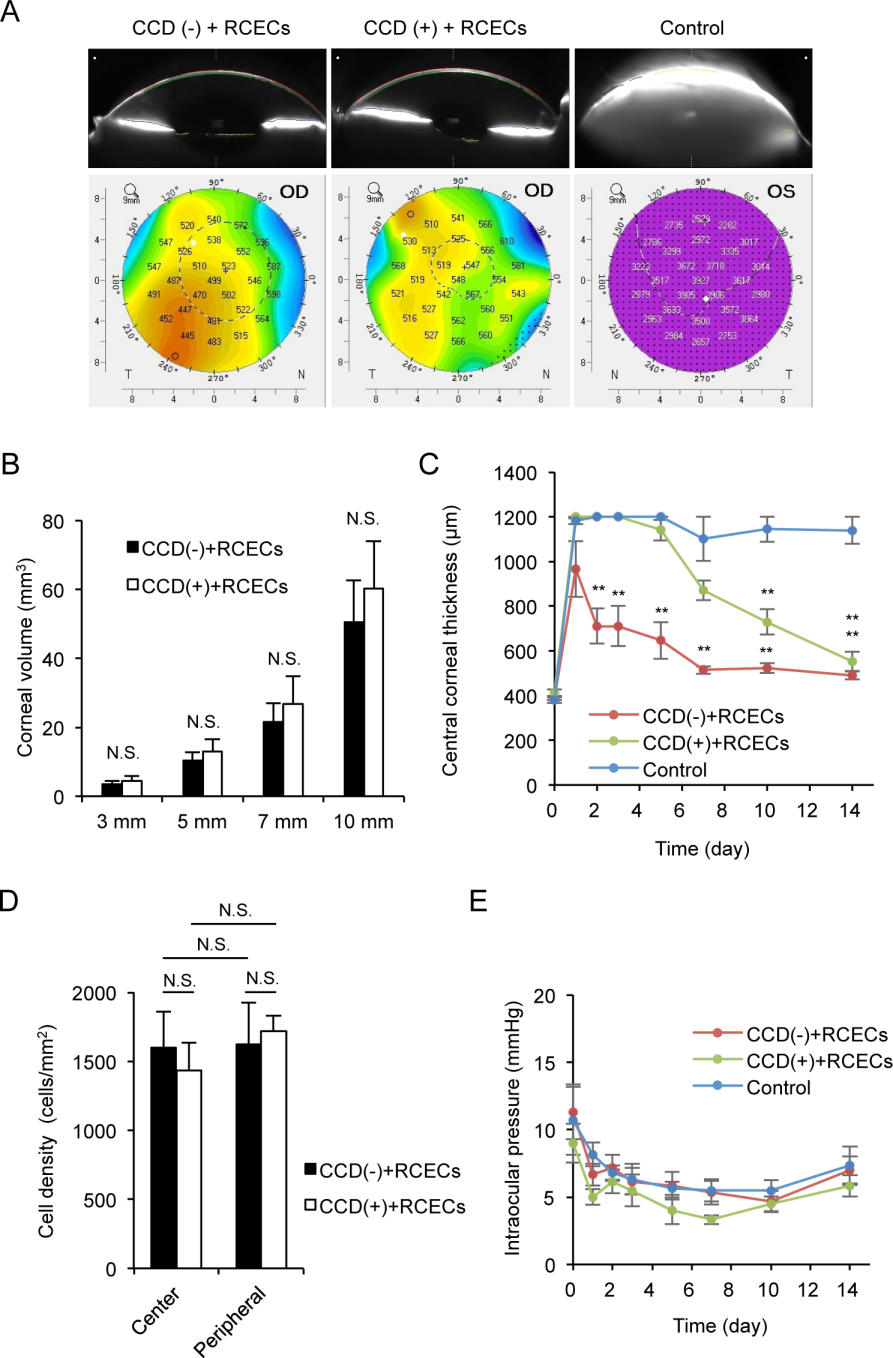
Assessment of the effect of circular descemetorhexis (CCD) on clinical parameters. (A) Scheimpflug images showed the restoration of an anatomically normal cornea in both the CCD (-) and CCD (+) models at 14 days after surgery. Control eyes showed corneal edema due to corneal endothelial dysfunction. A color map of corneal thickness showed that corneal thickness was thinner in both the CCD (-) and CCD (+) models when compared to the control. (B) The corneal volume was similar, when measured with a Pentacam^TM^ instrument at 3, 5, 7, and 10 mm diameters, in both the CCD (-) and CCD (+) models (n = 6). The corneal volume of the control group is not shown, as it was not evaluated with the Pentacam^TM^ instrument due to severe corneal edema. N.S. indicates no statistical significance. (C) The central corneal thickness was evaluated with an ultrasound pachymeter for 2 weeks after rabbit corneal endothelium cell (RCEC) injection. The eyes of the CCD (-) and CCD (+) models showed recovery of the central corneal thickness to an almost normal range, whereas this thickness did not recover in the controls. However, recovery of corneal thickness was slower in the CCD (+) model than in the CCD (-) model (n = 6). **P < 0.01, *P < 0.05. (D) Cell density of the regenerated corneal endothelium was determined by analyzing immunofluorescence staining images using ImageJ software. The average cell density of the restored corneal endothelium was similar for the CCD (-) and CCD (+) models in both the central and peripheral areas (n = 6). N.S. indicates no statistical significance. (E) Intraocular pressure (IOP) was evaluated with a Tonovet^®^ instrument, and no abnormal IOP elevation was observed in any of the groups (n = 6).

Scheimpflug images obtained with a Pentacam^TM^ instrument demonstrated anatomically normal corneas both in CCD (-) and CCD (+) models. Control eyes showed corneal edema due to corneal endothelial dysfunction (Fig 5A). A color map of corneal thickness showed that corneal thickness was obviously thinner in both the CCD (-) and CCD (+) models compared to the controls. The corneal volume for 3, 5, 7, and 10 mm diameters was similar for both the CCD (-) and CCD (+) models (Fig 5B). The central corneal thickness, evaluated with an ultrasound pachymeter, was significantly reduced in both the CCD (-) and CCD (+) models, when compared to controls, at 7, 10, and 14 days. The CCD (-) and CCD (+) models also showed recovery of the central corneal thickness to almost the normal range, whereas no recovery was observed in the controls. However, this recovery of corneal thickness was slower in the CCD (+) model than in the CCD (-) model (Fig 5C). The average cell density of restored corneal endothelium in the central area was 1602±241 cells/mm^2^ in the CCD (-) model and 1435±202 cells/mm^2^ in the CCD (+) model 14 days after surgery. In the peripheral area, the average cell density was 1625±302 cells/mm^2^ in the CCD (-) model and 1718±114 cells/mm^2^ in the CCD (+) model 14 days after surgery. These differences in cell density were not statistically significant (Fig 5D). Furthermore, no IOP elevation was observed in any of the groups (Fig 5E).

## Discussion

The prevalence of FECD in the United States is thought to be 4% of the population aged over 40 years [27], and this high prevalence makes FECD a leading cause of corneal transplantation. Indeed, the Eye Bank Association of America reported that FECD was the most common indication for corneal transplantation (49.2% the of corneal endothelial keratoplasty and 3.0% of penetrating keratoplasty procedures) utilizing corneas provided by U.S. eye banks [28]. Likewise, Gain et al., who performed a global survey of corneal transplantation by a systematic review of published literature, reported that the top indication of corneal transplantation was FECD (39% of all corneal transplantations) [29]. Given that FECD is a leading cause of corneal transplantation, it will be the most common cause for use of cell-based therapy when cultured human CECs are approved by the authorities in various countries in the future.

We obtained approval from the Japanese Ministry of Health, Labour, and Welfare to initiate clinical research into cell-based therapy for treating corneal endothelial decompensation. The inclusion criteria for this clinical research were: 1) the patient is diagnosed with corneal endothelial decompensation, 2) the best spectacle-corrected visual acuity is under 20/40, 3) the central corneal thickness is greater than 630 μm with corneal epithelial edema, and 4) corneal endothelial cell density is unmeasurable or lower than 500 cells/mm^2^ (Clinical trial registration: UMIN000012534, https://upload.umin.ac.jp/cgi-open-bin/ctr/ctr.cgi?function=brows&action=brows&type=summary&recptno=R000014592&language=E) [18]. Any types of original diagnoses resulting in corneal decompensation, such as FECD, corneal endothelial damage by various intraocular surgery, or eye trauma, are included in this clinical research. Although we are currently collecting clinical data, we have preliminary evidence that confirms that injection of cultured human CECs, only the removal of the pathological corneal endothelium but not Descemet’s membrane, results in regeneration of the corneal endothelium and restoration of a transparent cornea, regardless of the original diagnosis, including FECD (manuscript in preparation).

Descemet’s membrane is the basement membrane of the corneal endothelium. Basement membranes are cell-adherent extracellular scaffolds located at the basal side of every epithelium and endothelium [30]. They are anchored to the cytoskeleton through receptors, and they also act as a signaling platform for various essential cell phenomena [30–33]. In various cell types, components of the basement membrane bind to their corresponding integrins to initiate signaling from the outside to the inside of the cells by recruiting cytoplasmic adaptor proteins, phosphorylating binding proteins, and binding adaptor proteins to the actin cytoskeleton [31–33]. Descemet’s membrane is composed of type VI, VIII, XII, XVII collagens, glycoproteins (fibronectin, laminin, and osteonectin), and proteoglycans (versican and agrin) [20, 25, 34]. We have demonstrated that laminin-511 and 521 are expressed in Descemet’s membrane and play an imporatant role in CEC adhesion and proliferation and in the maintenance of functions though binding to integrins α3β1 and α6β1 [35]. Accumulating evidence now indicates an essential role for the basement membrane in cell fate, leading to the assumption that Descemet’s membrane will enhance engraftment of injected CECs in cell-based therapy.

Prior to initiating our clinical research, we performed numerous experiments using animal models to evaluate therapeutic effect and safety [16, 17, 24]. In terms of the method for removing the pathological corneal endothelium, we tried two methods: 1) remove only the corneal endothelium but not Descemet’s membrane and 2) remove the corneal endothelium with Descemet’s membrane by descemetorhexis. In rabbit and monkey corneal endothelial dysfunction models, we confirmed that the corneal endothelium was always restored when Descemet’s membrane was not removed [16, 17, 24]. By contrast, the success rate of restoration of a transparent cornea was reduced, the healing time to exhibit a transparent cornea was lengthened, and the cell density of regenerated corneal endothelium was lower in the animal models with Descemet’s membrane removal than in those without Descemet’s membrane removal (data not shown).

In the current study, we evaluated the feasibility of performing a small descemetorhexis of the optical zone. Our thinking was that removal of Descemet’s membrane in the optical zone is important for visual quality, while the remaining Descemet’s membrane might improve the fate of injected CECs. We showed that the corneal endothelium is regenerated directly onto the corneal stroma (the area of Descemet’s membrane removal) and corneal transparency was restored. However, recovery of central corneal thickness was slower when Descemet’s membrane was removed. We speculated that removal of Descemet’s membrane induced a greater severity of stromal edema than occurred when Descemet’s membrane was not removed, if the cell adhesion and the regeneration of corneal endothelium were similar in both the Descemet’s membrane removal model and the Descemet’s membrane non-removal model. Another possible explanation is that the corneal stroma is not an ideal substrate for adhesion of injected CECs or for recovery of CEC function when compared to the basement membrane. Even though the recovery time was longer, the final cell density and central corneal thickness, which are widely accepted as the most important clinical parameters of the corneal endothelium, were as good in the rabbit model with Descemet’s membrane removal as in the model without Descemet’s membrane removal at 14 days after surgery. Our study provides preclinical data showing that a small descemetorhexis of the optical zone may be a surgical option for cell based-therapy for the treatment of FECD.

In conclusion, we have demonstrated that a small descemetorhexis, in combination with cell-based therapy, is feasible to further improve visual quality after cultured CEC injection. Future randomized clinical trials of cell-based therapy for FECD, conducted with or without small descemetorhexisis, will be necessary to optimize the surgical protocol.

## Supporting information

S1 File Data set of Fig 5B–5E. (XLSX)
